# Missing links in understanding redox signaling via thiol/disulfide modulation: how is glutathione oxidized in plants?

**DOI:** 10.3389/fpls.2013.00477

**Published:** 2013-11-25

**Authors:** Marie-Sylviane Rahantaniaina, Andrée Tuzet, Amna Mhamdi, Graham Noctor

**Affiliations:** ^1^Institut de Biologie des Plantes, Université Paris-SudOrsay, France; ^2^Institut National de Recherche Agronomique, UMR Environnement et Grandes CulturesThiverval-Grignon, France

**Keywords:** oxidative stress, hydrogen peroxide, dehydroascorbate, glutathione *S*-transferase, glutaredoxin

## Abstract

Glutathione is a small redox-active molecule existing in two main stable forms: the thiol (GSH) and the disulphide (GSSG). In plants growing in optimal conditions, the GSH:GSSG ratio is high in most cell compartments. Challenging environmental conditions are known to alter this ratio, notably by inducing the accumulation of GSSG, an effect that may be influential in the perception or transduction of stress signals. Despite the potential importance of glutathione status in redox signaling, the reactions responsible for the oxidation of GSH to GSSG have not been clearly identified. Most attention has focused on the ascorbate-glutathione pathway, but several other candidate pathways may couple the availability of oxidants such as H_2_O_2_ to changes in glutathione and thus impact on signaling pathways through regulation of protein thiol-disulfide status. We provide an overview of the main candidate pathways and discuss the available biochemical, transcriptomic, and genetic evidence relating to each. Our analysis emphasizes how much is still to be elucidated on this question, which is likely important for a full understanding of how stress-related redox regulation might impinge on phytohormone-related and other signaling pathways in plants.

## Introduction

Arabidopsis cannot develop past the embryonic stage without glutathione (Cairns et al., 2006), a multifunctional tripeptide thiol found in the cells of most organisms. Although this small molecule has diverse roles in defense and metabolism, a key function is in redox homeostasis (Foyer and Noctor, 2011). This protective role notably involves acting in the metabolism of oxidants such as reactive oxygen species (ROS) and buffering protein thiol groups against excessive oxidation. Like most other biological roles of glutathione, these functions depend on the redox-active cysteine residue.

While the cysteine sulphur can exist in several redox states, the key stable forms are the thiol found in reduced glutathione (GSH) and the disulphide found in GSSG. Other factors remaining constant, the concentrations of the two forms modulate the effective redox potential of the couple according to the relationship [GSH]^2^/[GSSG] (Meyer, 2008). When plants are growing in unchallenging conditions, in which GSH-oxidizing compounds are kept relatively low by cellular antioxidant systems, the glutathione redox potential in subcellular compartments such as the cytosol, chloroplast, and mitochondria is maintained at highly reducing values. Redox-sensitive green fluorescent proteins (roGFP) have been developed as *in vivo* probes for cell thiol-disulfide status. Although it cannot be completely excluded that other thiols may influence their status *in vivo*, the *in vitro* specificity of roGFP oxidoreduction suggests that they report mainly on the glutathione redox potential (Meyer et al., 2007). Analyses using these probes have measured redox potentials in the cytosol, chloroplasts and mitochondria that are close to −320 mV, the midpoint potential of NADPH (Meyer et al., 2007; Schwarzländer et al., 2008; Jubany-Mari et al., 2010). If these values faithfully reflect the glutathione redox potential, they imply that the GSH:GSSG ratio is well over 1000. Thus, while total glutathione concentrations are typically in the 1–10 mM range (Queval et al., 2011), GSSG concentrations in unchallenging conditions may be 10^3^–10^6^ times lower in compartments that contain significant activities of GR.

When plants are subject to suboptimal conditions, GSSG can accumulate to higher levels. This phenomenon is observed in extracts of plants exposed to various abiotic and biotic stresses (Edwards et al., 1991; Sen Gupta et al., 1991; Vanacker et al., 2000; Bick et al., 2001; Gomez et al., 2004). Based on studies of plants deficient in enzymes such as ascorbate peroxidase (APX) and catalase (CAT), accumulation of GSSG is quite closely related to the intracellular availability of H_2_O_2_ (Rizhsky et al., 2002; Mhamdi et al., 2010a), which is expected to be enhanced in stress conditions.

The principal reactions and proteins responsible for reducing GSSG to GSH in plants are relatively well characterized. In Arabidopsis, two genes each encode dual-targeted glutathione reductases (GR), and this is sufficient to explain the presence of GR activity in the chloroplasts, mitochondria, cytosol, and peroxisomes (Chew et al., 2003; Kataya and Reumann, 2010). Given that GR has a *K*_M_ value for NADPH below 10 μM (Edwards et al., 1990), conversion of GSSG to GSH is unlikely to be limited by reductant. Moreover, loss of function of *GR1*, encoding the cytosol/peroxisome enzymes, causes only moderate GSSG accumulation in leaf tissue, an observation explained by the existence of an auxiliary GSSG-reducing activity ensured by cytosolic NADPH-thioredoxin (TRX) systems (Marty et al., 2009). However, GR1 becomes more important in oxidative stress conditions (Mhamdi et al., 2010b; Dghim et al., 2013).

In contrast to GSSG reduction, the reactions that are most important in converting GSH to GSSG are less clear and, potentially, more complex. Since GSSG accumulation is not only a useful biochemical marker for oxidative stress in plants, but may also be of functional importance in transmitting signals triggered by increased H_2_O_2_ (Han et al., 2013a,b), the aim of the discussion below is to present an overview of current knowledge on the reactions that could be responsible for this phenomenon.

## Candidate pathways

### Enzyme-independent oxidation

Because GSH oxidation is strongly dependent on deprotonation to the thiolate form (GS−), electron transfer is pH-dependent. The p*K*a of the GSH thiol is about 9.0. Thus, only about 1% of GSH thiols will be deprotonated at any one moment in the cytosol (pH 7.2). This percentage will be even less in more acidic compartments such as the vacuole or apoplast, although GSH concentrations are relatively low at these locations. In the chloroplast, the chemical reactivity of GSH will be favored in the light compared to the dark because photosynthetic electron transport drives alkalinisation of the stroma. Chemical oxidation of GSH can therefore be influenced by physiologically relevant changes in pH. Decreases in proton concentration will also decrease the glutathione redox potential for a given value of [GSH]^2^/[GSSG].

The nucleophilic properties of GS- mean that it can react with a wide spectrum of electrophiles. In some cases, this will not lead to oxidation to GSSG or other disulfide but rather formation of a stable *S*-conjugate with various compounds (Dixon and Edwards, 2010). Such GS-conjugates are generally transported by ATP-dependent pumps (ABCC proteins) to the vacuole, where the constituent amino acids of the glutathione moiety are recycled (Martinoia et al., 1993; Lu et al., 1998; Grzam et al., 2006). Among the molecules able to oxidize GSH to produce GSSG are ROS and dehydroascorbate (DHA), the stable non-radical product of ascorbate oxidation. Rate constants for reactions with some of these compounds are shown in Table 1. Glutathione reacts with singlet oxygen and superoxide at rates similar to other molecules with recognized antioxidant properties, such as ascorbate and phenolic compounds. However, both glutathione and ascorbate react appreciably slower with singlet oxygen than tocopherols and, especially, carotenoids (Table 1). While the reaction between GSH and the hydroxyl radical is very fast, this powerful oxidant also reacts rapidly with numerous other metabolites that are present in the cellular environment at higher concentrations than GSH, such as ascorbate and sugars (Table 1). Thus, other compounds might be expected to compete effectively with GSH in the scavenging of both singlet oxygen and the hydroxyl radical. As for other non-enzymatic thiols, and ascorbate, the chemical reaction with H_2_O_2_ is very slow. Superoxide and DHA are therefore the most likely of the molecules shown in Table 1 to contribute to uncatalyzed production of GSSG *in vivo*. This conclusion receives some support from kinetic modeling studies (Polle, 2001).

**Table 1:** **Rates of nonenzymatic reactions between glutathione and various oxidants**.

**Oxidant**	**Metabolite**	**k (M^−1^s^−1^)**
Singlet oxygen	Glutathione^a^	2 × 10^6^
	Ascorbate^a^	1 × 10^7^
	β-Carotene^b^	1.4 × 10^10^
	α-Tocopherol^b^	3 × 10^8^
Superoxide	Glutathione^a^	7 × 10^5^
	Ascorbate^a^	2 × 10^5^
	Kaempferol^c^	5.5 × 10^5^
	Quercetin^c^	0.9 × 10^5^
Hydroxyl radical	Glutathione^f^	8.1 × 10^9^
	Ascorbate^f^	1.5 × 10^9^
	Kaempferol^c^	4.6 × 10^9^
	Quercetin^c^	4.3 × 10^9^
	Glucose^f^	4.0 × 10^9^
	Sucrose^f^	8.9 × 10^9^
	Galactinol^f^	7.8 × 10^9^
Hydrogen peroxide	Glutathione^d^	0.9
	Ascorbate^e^	2
	Cysteine^d^	2.9
	Thioredoxin^d^	1.1
Dehydroascorbate	Glutathione^g^	1 × 10^5^

*For comparative purposes, rate constants are also shown for ascorbate and other metabolites*.

References:

a Asada and Takahashi, 1987;

b Di Mascio et al., 1989;

c Bors et al., 1990;

d Winterbourn, 2013;

e Polle, 2001;

f Nishizawa et al., 2008;

g *Hausladen and Kunert, 1990. Values for thioredoxin are for the E.coli protein*.

### The ascorbate-glutathione pathway

**Figure 1:**
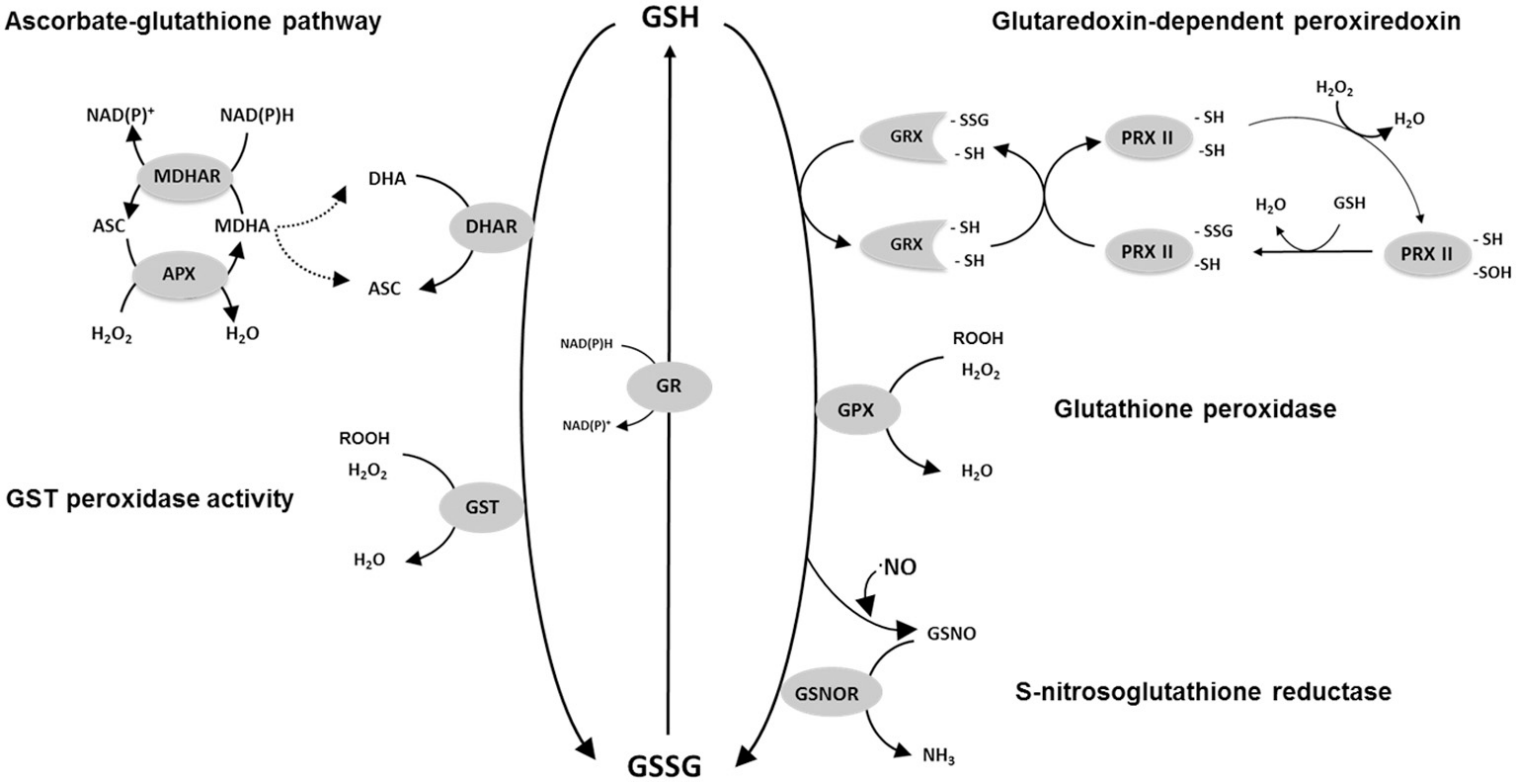
**Scheme of some of the possible reactions involved in GSH oxidation in plants**. Not all possible reactions or reaction mechanisms are shown. For reasons of clarity, stoichiometries are not depicted. For PRXII a proposed regeneration mechanism of Prx IIE using the GSH/Grx system is shown according to Gama et al. (2008). Abbreviations: GSH, reduced glutathione; GSSG, glutathione disulphide; GR, glutathione reductase; DHAR, dehydroascorbate reductase; APX, ascorbate peroxidase; MDHAR, monodehydroascorbate reductase; ASC, ascorbate; DHA, dehydroascorbate; MDHA, monodehydroascorbate; H_2_O_2_, hydrogen peroxide; GST, glutathione *S*-transferase, GRX, glutaredoxin; PRX, peroxiredoxin; -SH, sulfhydryl (thiol) group; -SSG, glutathionylated protein Cys residue; GSNO, S-nitrosoglutathione; GSNOR, GSNO reductase; GPX, glutathione peroxidase.

The slow chemical reaction of GSH with H_2_O_2_ contrasts with the rapid reaction with DHA (Table 1). This is the key observation underlying the importance ascribed to the close redox coupling of ascorbate and glutathione pools *in vivo*, which allows glutathione to play an indirect role in H_2_O_2_ reduction as part of a reaction sequence that ultimately depends on electrons derived from NAD(P)H and/or ferredoxin (Figure 1). Thus, APX reduces H_2_O_2_ to water, yielding monodehydroascorbate (MDHA) as an unstable initial product. MDHA that is not rapidly reduced can dismutate to ascorbate and DHA, which can then be reduced by GSH with the concomitant production of GSSG (Figure 1).

While GSH can chemically reduce DHA at high rates, the reaction is accelerated significantly by DHA reductases (DHAR; Foyer and Halliwell, 1977). These enzymes have been purified from several species (Hossain and Asada, 1984; Dipierro and Borranccino, 1991; Urano et al., 2000). In Arabidopsis, at least three *DHAR* genes are expressed. Although they are not able to catalyze glutathione *S*-conjugation reactions at significant rates, they are considered to belong to the glutathione *S*-transferase (GST) superfamily (Dixon and Edwards, 2010). The difference in the activities of DHARs and most classes of GST is explained by the presence of a cysteine in place of serine at the active sites of DHARs (Dixon et al., 2002).

### Glutathione peroxidase (GPX)

Although GSSG could be generated as a product of GSH-dependent DHA reduction, genomics has revealed the complexity of the plant antioxidative system and identified several GSH-dependent enzymes that may play more direct roles in peroxide metabolism. GPX has long been known to be a player in H_2_O_2_ metabolism in mammalian cells, but only began to be seriously studied in plants following the description of sequences homologous to the animal enzymes in the 1990s. Described as glutathione hydroperoxidases, the plant GPXs are distinguished from animal GPXs in having an active site cysteine in place of selenocysteine (Eshdat et al., 1997), even though both selenocysteine- and cysteine-dependent GPXs are found in unicellular algae such as *Chlamydomonas* (Dayer et al., 2008). Genome sequencing has shown that plant GPXs are encoded by several genes (8 in Arabidopsis). Despite their current nomenclature, several independent studies have shown that the encoded enzymes prefer TRX as reductant and have comparatively low activity against GSH (Herbette et al., 2002; Iqbal et al., 2006; Navrot et al., 2006). Thus, they might be considered to be TRX-dependent peroxiredoxins, and so not strong candidates to account for GSSG accumulation *in vivo*. However, they are included in Figure 1 because it cannot as yet be discounted that they make some contribution to the GSH oxidation that occurs during stress.

### Glutathione S-transferases (GST)

The *GST* superfamily is composed of 55 genes in Arabidopsis, including the *DHAR* sequences mentioned above (Dixon and Edwards, 2010). As well as the DHARs, the family is divided into several classes (zeta, theta, TCHQD, phi, tau, lambda), with the last three being specific to plants. The most numerous are the phi and tau classes, composed of 13 and 28 genes, respectively (Supplemental Table 1). Proteins that catalyze the classical conjugase reaction using GSH are found in several classes. At least some GSTs can also use GSH to reduce organic hydroperoxides (Cummins et al., 1999; Figure 1). Studies on the Arabidopsis proteins have revealed that several classes of GST include enzymes with both conjugase and peroxidase activities (Dixon et al., 2009; Dixon and Edwards, 2010). Enzymes of the lambda class are unusual in that they do not catalyze conjugase reactions. Like DHARs, they have an active-site cysteine and function as monomers (Dixon et al., 2002). They may generate GSSG by catalyzing the reduction of small molecules or, possibly, the deglutathionylation of protein cysteine residues (Dixon et al., 2002; Dixon and Edwards, 2010).

### Peroxiredoxins (PRX)

These enzymes are classed into several types: 2-cys PRX, 1-cys PRX, type II PRX, and PRX Q (Dietz et al., 2002). The first to be studied in plants was chloroplastic 2-Cys PRX, which can be regenerated by specific thioredoxins or by an NADPH-thioredoxin reductase (Dietz, 2003; Collin et al., 2004; Pulido et al., 2010). However, plants also contain several type II PRX that, once oxidized by peroxides or other compounds, can oxidize glutathione, particularly *via* glutaredoxins (GRX; Figure 1). Interactions between PRXII and GRX have been studied at the biochemical level in poplar and *Arabidopsis* (Rouhier et al., 2002; Bréhélin et al., 2003; Couturier et al., 2011; Riondet et al., 2012). PRXII are encoded by five expressed genes in Arabidopsis. While information is emerging, the identification of the GRXs that couple their re-reduction to GSH oxidation *in vivo* remains incomplete (Rouhier, 2010).

### Other possibilities

The reactions outlined above are not intended to be exhaustive. Numerous other routes could allow oxidation of GSH to GSSG. A comprehensive treatment of all of these is beyond the scope of this discussion. Among other possibilities of note is the reaction catalyzed by *S*-nitrosoglutathione (GSNO) reductase (Figure 1), which can produce GSSG from GSH and GSNO. Although this enzyme is receiving considerable attention for its role in various physiological functions (Sakamoto et al., 2002; Díaz et al., 2003; Barroso et al., 2006; Kwon et al., 2012), its capacity relative to enzymes such as DHAR is unclear. Adenosine phosphosulfate reductase (APR), a key chloroplastic enzyme in sulphate reduction, uses GSH as electron donor (Bick et al., 1998). The capacity of this enzyme is relatively low, although the activity may be stimulated by enhanced expression and post-translational activation in oxidative stress conditions, notably to produce cysteine for glutathione synthesis (Bick et al., 2001; Queval et al., 2009).

At least one type of plant methionine sulphoxide reductase activity may be coupled to GSH oxidation *via* glutathione-linked GRX (Tarrago et al., 2009). This enzyme regulates the oxidation state of protein methionine residues but based on its turnover rates (Tarrago et al., 2009), it is unlikely to make an appreciable contribution to increases in GSSG during oxidative stress. One interesting mechanism that could potentially contribute has been described as “proteome-dependent glutathione peroxidase” (Zaffagnini et al., 2012). This process, which could be stimulated under conditions of stress, envisages a chloroplastic sequence of reactions involving H_2_O_2_-triggered *S*-glutathionylation of diverse available protein cysteine residues, followed by regeneration of the free cysteines by glutathione-dependent GRX (Zaffagnini et al., 2012). The net result would be reduction of H_2_O_2_ to two water molecules with oxidation of 2 GSH to GSSG, i.e., a GSH peroxidase reaction. Such a sequence may share mechanistic features with the PRXII-GRX pathway shown in Figure 1, a principal difference being that H_2_O_2_ would not react with a specific catalytic cysteine but rather in a more general way with free and reactive chloroplast protein cysteines. As yet, the physiological significance of this process is difficult to evaluate, although it has been noted that the abundance of potentially reactive cysteines in chloroplast proteins is far from negligible (Zaffagnini et al., 2012).

## Subcellular compartmentation

**Figure 2:**
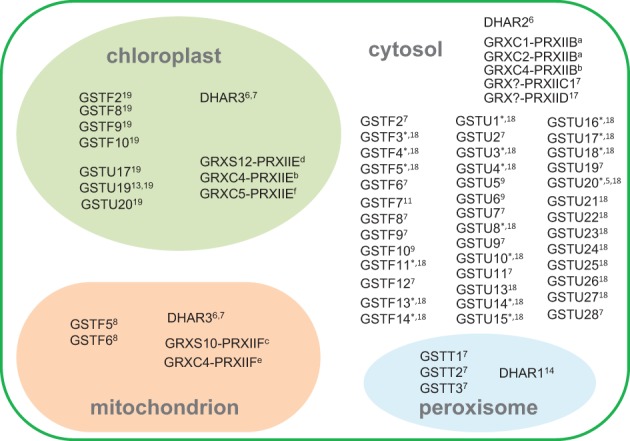
**Subcellular compartmentation of GSH-oxidizing enzymes, based on available information**. Numbers and “*” indicate references given in Supplemental Table 1 except for the following, indicated in the figure by letters in superscript: ^a^Riondet et al., 2012; ^b^Bréhélin et al., 2003; ^c^Finkemeier et al., 2005; ^d^Gama et al., 2008; ^e^Rouhier et al., 2005; ^f^Couturier et al., 2011. Abbreviations as in Figure 1 legend. GRX-PRX partnerships are indicated for proteins shown to functionally interact in peroxidase activity, but these proteins may also interact with other partners.

Supplemental Table 1 presents a list of Arabidopsis genes involved in the pathways shown in Figure 1. Given the relative concentrations of different ROS, the battery of H_2_O_2_-metabolizing enzymes potentially linked to glutathione, and the marked changes in glutathione status when other H_2_O_2_-metabolizing enzymes such as catalase are down-regulated, the main focus of the following discussion concerns the enzymes that could be important in linking H_2_O_2_ or related peroxides to GSH oxidation to GSSG. Based on the above discussion, we suggest that the major candidates to perform this function are (1) DHARs, (2) GSTs, and (3) GRX-PRXII. The subcellular compartmentation of Arabidopsis proteins within these families is summarized in Figure 2.

While DHAR1 has been localized to the peroxisomes, DHAR2 is cytosolic and *DHAR3* encodes a dual-addressed chloroplast/mitochondrial enzyme (Figure 2). These three genes are therefore sufficient to explain the presence of the ascorbate-glutathione cycle in these compartments (Foyer and Halliwell, 1977; Jiménez et al., 1997). Three type II PRX (PRXIIB, C, D) are found in the cytosol, with PRXIIE and PRXIIF located in the chloroplast and mitochondrion, respectively (Figure 2; Rouhier and Jacquot, 2005; Tripathi et al., 2009).

Many GSTs are located in the cytosol, but several of these have also been detected in other compartments such as the chloroplast (Figure 2; Supplemental Table 1). The only types of GST that are not thought to be found in the cytosol are the theta class, encoded by three genes that direct the proteins to the peroxisome (Dixon et al., 2009). The GFP-fusion proteins of GSTU12 and GSTT3L were found to be localized in the nucleus (Dixon et al., 2009).

## Evidence for the importance of the different pathways

### Biochemical data

#### DHA as oxidant

On the basis of modeling of ROS metabolism in the chloroplast, it was suggested that the ascorbate pool could operate largely independently of glutathione because most of the MDHA produced would be efficiently reduced to ascorbate, entailing the formation of little DHA (Polle, 2001). It was concluded that the chemical reaction with GSH would suffice to reduce the small amounts of DHA produced (Polle, 2001). It is possible that this situation is specific to the illuminated chloroplast.

Both the chemical and enzyme-dependent reduction of DHA can be easily detected *in vitro* by following the GSH-dependent production of ascorbate in the absence and presence of crude protein extract. The total enzyme-dependent leaf activity that can be measured in standard assay conditions is around 0.2–0.5 μmol.mg^−1^ total protein.min^−1^, which is typically about twice the total leaf GR activity but somewhat lower than extractable APX (e.g., Sen Gupta et al., 1993; Mhamdi et al., 2010b).

A protein purified from spinach by classical biochemical procedures, considered to be a cytosolic DHAR, had *K*_M_ values of 2.5 mM and 70 μM for GSH and DHA, respectively (Hossain and Asada, 1984). Analysis of a purified chloroplast protein produced similar values, although with a somewhat higher affinity for GSH (Shimaoka et al., 2000). Subsequent studies of recombinant DHARs from Arabidopsis, poplar, and rice produced similar maximal activities and *K*_M_ values to these preparations, although the organellar forms in Arabidopsis have a significantly lower affinity for GSH (Table 2). With the exception of values reported for the Arabidopsis enzymes, the *K*_M_ values for GSH are below or close to *in vivo* concentrations (Queval et al., 2011). Much of the DHA that can be measured in unstressed plant tissues is probably apoplastic (Foyer and Noctor, 2011) and concentrations are probably low in compartments that contain GSH (Polle, 2001). If so, oxidative stress-induced changes in DHA could be a major factor contributing to accelerated activity *in vivo*, at least in some compartments. Under conditions favoring accumulation of H_2_O_2_, a second factor that could come into play is increases in DHAR abundance. The expression of some *DHAR* genes is increased by oxidative stress (discussed further in the next section).

**Table 2:** **Kinetic properties of dehydroascorbate reductases from several plant species**.

**Name/Subcellular localization**	**Max activity**	**GSH**			**DHA**		
		***K*_M_**	***k*_cat_ (/10^4^)**	***k*_cat_/*K*_M_ (/10^4^)**	***K*_M_**	***k*_cat_ (/10^4^)**	***k*_cat_/*K*_M_ (/10^4^)**
**PURIFIED FROM SPINACH LEAVES**							
Cyt^a^	370	2.5	–	–	0.07	0.01	–
Chp^b^	360	1.1	–	–	0.07	0.03	–
Chp^c^	–	1.1	–	0.52	0.05	0.05	0.9
**RECOMBINANT ARABIDOPSIS ENZYMES^d^**							
DHAR1 Per	936	10	–	–	0.26	–	–
DHAR2 Cyt	120	–	–	–	–	–	–
DHAR3 Chp/Mit	264	10	–	–	0.50	–	–
**RECOMBINANT POPLAR ENZYMES^e^**							
DHAR1 Chp	53	3.8	9.9	2.7	0.07	1.3	18.8
DHAR2 Cyt	50	2.3	5.4	2.4	0.23	1.8	7.7
DHAR3 Cyt	38	2.5	4.1	1.7	0.48	2.1	4.4
**RECOMBINANT RICE ENZYME^f^**							
DHAR1 Cyt	350	1.0	–	–	0.35	–	–

*Values are expressed in mM (K_M_), s^−1^ (k_cat_,), mM^−1^s^−1^ (k_cat_/K_M_), and μmol. min^−1^mg^−1^protein (maximal activity). Chp, chloroplast. Cyt, cytosol. Mit, mitochondrion. Per, peroxisome*.

References:

a Hossain and Asada, 1984;

b Shimaoka et al., 2000;

c Shimaoka et al., 2003;

d Dixon et al., 2002;

e Tang and Yang, 2013;

f *Amako et al., 2006*.

#### GST activities

Although they have hydroperoxide activity, GSTs generally use H_2_O_2_ only at low rates (Mannervik, 1985). The physiological oxidants used by the different GSTs in plants remain in many cases to be identified, and studies of their biochemical activities frequently use the artificial substrate, cumene hydroperoxide. Specific GSTs have been purified from several species and their activities as conjugases or peroxidases compared (DeRidder et al., 2002; Cummins et al., 2003; Park et al., 2005; Nutricati et al., 2006; Yang et al., 2009). Such studies reveal that the *K*_M_ values of GSTs for GSH in conjugase reactions are generally below 2 mM. Some studies have compared the peroxidatic competence of several GSTs from the same species (Wagner et al., 2002; Dixon et al., 2009). Most notably, the detailed study of Dixon et al. (2009) reported that GSTs showing peroxidase activity are numerous and not limited to any class. Of 38 theta, phi, and tau class GSTs tested for GSH peroxidase activity against short-chain organic peroxides, only six were found to have undetectable activity (Table 3). Of the 32 with detectable peroxidase activity, most were also able to catalyze GSH conjugation to one or both of two model substrates (Dixon et al., 2009).

**Table 3:** **Arabidopsis recombinant glutathione *S*-transferases shown to have GSH peroxidase activity *in vitro***.

**PEROXIDATIC ACTIVITY**					
T1 > U25 > T3 > T2 > U8 > U17 > U24 > F6 > F8 > U6 > U16 > U5 > F2 > F9 > U18 > U3 > U19 > U1 > U22 > F7 > U4 > F3 > U20 = U23 > U10 = U26 > U28 = U2 > U9 > U7 > U21 > U13					
**PEROXIDATIC ACTIVITY NOT DETECTED**					
F5	F14	U11	U12	U14	U27
**PEROXIDATIC ACTIVITY NOT TESTED**					
F4	F13	F10	F11	F12	U15

*The enzymes for which peroxidatic activity was detected are listed in descending order of the activity measured on a unit protein basis against cumene hydroperoxide (Dixon et al., 2009)*.

Although 32 GSTs showed peroxidase activity *in vitro*, their specific activities varied considerably, the most active (GSTT1) having rates ~600-fold higher than the least active (GSTU13). The relatively small theta class was the only one whose members were all found to be competent in GSH peroxidation. Together with the tau-type U25, these three GSTs showed the highest specific peroxidase activity (Table 3). Moreover, the theta class enzymes were active not only against cumene hydroperoxide but also against long-chain (C_18_) fatty acid peroxides. This contrasted with U25, which was highly active only against the model peroxide (Dixon et al., 2009). Interestingly, the theta GSTs were shown to be localized in the peroxisomes (Figure 2), organelles that can have high rates of both peroxide generation and fatty acid metabolism.

#### Glutaredoxin-linked peroxiredoxins

The activity of recombinant poplar mitochondrial PRXIIF against H_2_O_2_ and organic peroxides was measured in the presence of GSH and/or GRX (Gama et al., 2007). With *tert*-butyl hydroperoxide as oxidant, a *K*_M_ of 260 μM was obtained for GSH. However, the activity was considerably stimulated by the additional presence of poplar GRX C4 (Gama et al., 2007). Analysis of the chloroplastic PRXIIE (Gama et al., 2008) revealed fairly similar properties, except that lower *K*_M_ peroxide and higher *k*_cat_ values produced somewhat higher catalytic efficiencies than for the mitochondrial protein (Table 4). In fact, the kinetic properties against peroxide of the glutathione-linked PRXIIE were very similar to 2-cys PRX, which is also chloroplastic but glutathione-independent (Horling et al., 2003; Bernier-Villamor et al., 2004; Rouhier et al., 2004a,b; Gama et al., 2007). A study of the mitochondrial PRXIIF from pea reported a similar *K*_M_ for H_2_O_2_ but significantly higher turnover values (Barranco-Medina et al., 2007). The *K*_M_ values for H_2_O_2_ of PRXII, 2-cys PRX and APX are quite similar, but the turnover rates of both types of PRX are significantly lower. Thus, *k*_cat_/*K*_M_ values for PRX are about 100-fold below those measured for chloroplastic APX or, in the case of the pea PRXIIF, over 10-fold lower (Table 4).

**Table 4:** **Kinetic chacteristics of glutaredoxin-dependent peroxiredoxins from poplar and pea**.

**Enzyme**		**Substrate**	**Peroxide**			**GSH**	**GrxC4**
			***K*_M_ (mM)**	***k*_cat_ (s^−1^)**	***k*_cat_/*K*_M_ (mM^−1^s^−1^)**	***K*_M_ (mM)**	***K*_M_ (μM)**
Poplar PRXIIF^a^	Mit	H_2_O_2_	0.07	0.38	5.3		
		*t*-BOOH	0.02	0.51	31.5	0.260	1.3
		CuOOH	0.33	0.39	1.2		
Pea PRXIIF^b^	Mit	H_2_O_2_	0.02	10.6	560	ND	ND
Poplar PRXIIE^c^	Chp	H_2_O_2_	0.02	0.57	26		
		*t*-BOOH	0.01	0.90	104	ND	0.51
Pea 2-Cys PRX^d^	Chp	H_2_O_2_	0.03	0.69	25	NA	NA
Spinach APX^e^	Chp	H_2_O_2_	0.03	290	9667	NA	NA

*For comparison, data are shown for 2-cys peroxiredoxin from pea (Pisum sativum) and ascorbate peroxidase (APX) from spinach (Spinacia oleracea). t-BOOH, tert-butyl hydroperoxide. CuOOH, cumene hydroperoxide. ND, not determined. NA, not applicable*.

References:

a Gama et al., 2007;

b Barranco-Medina et al., 2007;

c Gama et al., 2008;

d Bernier-Villamor et al., 2004;

e *Nakano and Asada, 1987*.

In terms of the capacity for GSH oxidation through GRX-PRXII compared to the ascorbate-glutathione pathway, it is interesting to compare the *k*_cat_ and *k*_cat_/*K*_M_ values of PRXII and DHAR obtained when the respective oxidants (peroxide and DHA) were varied. Based on available data, the parameters for DHAR are about 20–1000-fold higher (compare Tables 2, 4). For equal amounts of protein, this indicates that DHAR should be more efficient, although the actual *in vivo* rates will be influenced by several factors, most obviously the relative abundance of the proteins and the *in vivo* concentrations of the respective oxidants in the compartments where the proteins are located.

Glutaredoxins can catalyze reduction of DHA *in vitro* (Wells et al., 1990). Arabidopsis GRXC1 and C2, which are competent in regeneration of PRXIIB, can also catalyze DHA reduction (Riondet et al., 2012). *K*_M_ values of the GRX for DHA were similar to DHAR but the *k*_cat_ values were only about 3 s^−1^ (Riondet et al., 2012), several orders of magnitude lower than the DHARs (Table 2).

### Gene expression during oxidative stress

**Figure 3:**
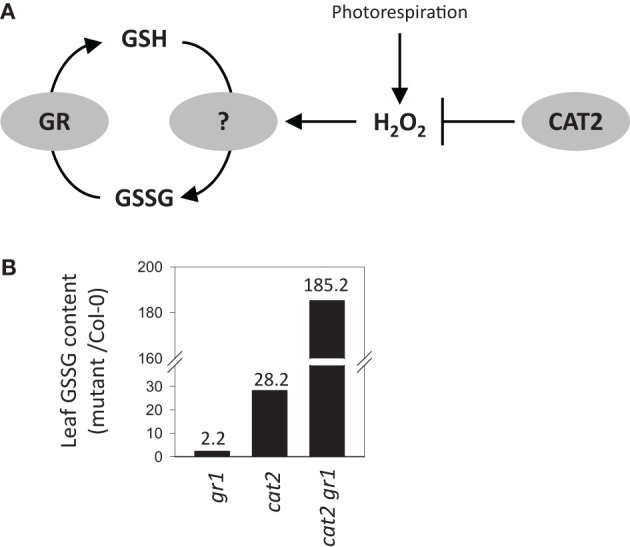
**Accumulation of GSSG relative to Col-0 in Arabidopsis knockout mutants for glutathione reductase 1 (*gr1*), catalase 2 (*cat2*), or both (*cat2 gr1*). (A)**Simple scheme showing the interplay between GR and H_2_O_2_-triggered oxidation in determining the balance between GSH and GSSG in the mutant systems. **(B)** Leaf GSSG contents in the three mutant lines relative to Col-0 controls. Fold-change compared to Col-0 GSSG contents are indicated above the bars.

There have been many transcriptomic analyses of plants undergoing oxidative stress, conditions in which oxidation of GSH is expected to be accelerated. However, the number of such studies that have included data on glutathione status is more limited. These notably include Arabidopsis mutants deficient in the major leaf form of catalase (CAT2), in which conditionally increased H_2_O_2_ availability through photorespiration drives reproducible changes in GSSG:GSH (Mhamdi et al., 2010a). These increases in leaf GSSG are also observed, to a lesser extent, in knockout mutants for GR1, and are particularly marked in *cat2 gr1* double mutants lacking both enzymes (Figure 3). However, the processes that are responsible for GSSG accumulation when CAT2 function is lost remain unclear.

**Figure 4:**
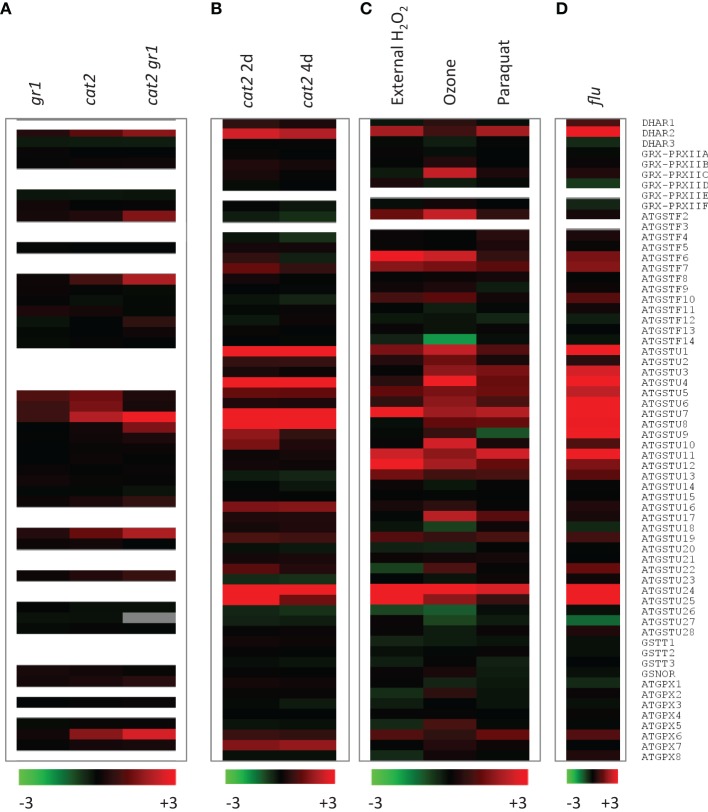
**Expression of candidate genes involved in glutathione oxidation in GSSG-accumulating and/or oxidative stress mutants and in conditions promoting oxidative stress**. Transcript abundance are shown as log_2_ values compared to control (wild-type or untreated). Experimental details are given in Supplemental Table 1. Red and green indicate induction and repression according to the scales shown at the bottom. White rows indicate absence of a corresponding probe set from the array chip. “GRX-PRXII” denotes the signals for the indicated PRX. **(A)** GSSG-accumulating lines shown in Figure 2 (*gr1*, *cat2*, *cat2 gr1*). Oxidative stress was induced in 3 week old plants (Mhamdi et al., 2010b). **(B)** Two independent datasets for *cat2* at two timepoints after onset of oxidative stress (2 and 4 days). Oxidative stress was induced in 5 week old plants (Queval et al., 2012). **(C)** Genevestigator data for chemically and environmentally induced oxidative stress (H2O2, paraquat and ozone). **(D)** Genevestigator data for the singlet oxygen-accumulating *flu* mutant (Laloi et al., 2007).

As a first step to investigating this question, we mined two Arabidopsis *cat2* microarray datasets for genes encoding the enzymes listed in Supplemental Table 1. The data on *cat2* were compared with responses to external H_2_O_2_, ozone, and paraquat (Figure 4C) and to results obtained for the *flu* mutant, which generates excess singlet oxygen in the chloroplast (Figure 4D). Experimental details for all these microarray studies are given in Supplemental Table 2. The two datasets in Figures 4A,B come from different experiments performed on leaf rosette material from plants of different age (Mhamdi et al., 2010b; Queval et al., 2012). They were also obtained using two different microarray chips, and some probe sets were present on only one of the chips (Figure 4: absence of corresponding probe sets is indicated by white rows). Moreover, key factors determining the measured response to oxidative stress are likely to be exposure time and stress intensity. As well as other differences in plant growth conditions, the data shown in the different parts of Figure 4 were obtained after different exposure times, ranging from 1 h (H_2_O_2_; Figure 4C) to 4 days (Figures 4A,B, right column).

For all the above reasons, some divergence is expected between responses observed in the different datasets. Despite this, a number of genes within each family responded quite similarly to the different oxidative stresses. Among the three DHARs, *DHAR2* was the most obviously responsive, being clearly induced in *cat2*, *flu*, and by external H_2_O_2_ or paraquat. Induction was less evident following ozone exposure (Figure 4). PRXIIC was the only GRX-linked PRX that was induced, though only in response to ozone. None of the *PRXII* genes were induced alongside GSSG accumulation in *cat2* (Figures 4A,B). In contrast, marked up-regulation of several *GST*s was observed in most of the datasets. GSTs that responded to most of the treatments included F6 and F7, as well as many tau types, with U1, U4, U5, U6, U7, U8, U24, and U25 showing a particularly clear induction. Indeed, U24 and U25 are among the most strongly *cat2*-induced genes on a fold-change basis (Queval et al., 2012). Interestingly, U11, U12, and U13 were induced by all treatments except in *cat2* and *cat2 gr1* (Figure 4, compare A and B with C and D). This could indicate that these GSTs are involved in early responses to oxidative stress. In contrast to several phi and tau *GST* genes, none of the theta types were induced in any condition, despite their documented high peroxidase activity (Table 3). Little or no induction of *GSNOR* was apparent, while among the *GPX* sequences, *GPX5*, *GPX6*, and *GPX7* were most obviously responsive, although some variation was observed between the experiments, including the two *cat2* datasets.

This comparison points to numerous candidates that could play a role in oxidizing glutathione. For several reasons, however, the expression data remain at best indicative. First, increased transcripts may not feed through to an increase in protein abundance. Second, transcriptomic analyses do not identify other possible regulatory mechanisms that may operate during oxidative stress at the post-transcriptional or post-translational levels. Third, the data of Figure 4 show fold-changes compared to wild-type: even if strongly induced at the protein level, low abundance enzymes may not make a marked contribution to GSH oxidation within the cellular context. Inversely, it is not possible to discount a role for an enzyme on the basis of lack of induction. Finally, even if transcript up-regulation feeds through to enhanced protein, some of the enzyme activities encoded by responsive genes shown in Figure 4 may not involve GSH oxidation. At least some of the GSTs are probably induced in connection with a conjugase function. For example, GSTF6 has been implicated in the synthesis of camalexin (Su et al., 2011), a phytoalexin that can accumulate strongly in *cat2* in oxidative stress conditions (Chaouch et al., 2010). As noted above, GPXs may mainly if not exclusively catalyze peroxidation using TRX, not GSH.

With the above caveats in mind, useful pointers can be obtained by combining information from biochemical competence and efficiency (Tables 2–4) with responsiveness at the transcript level (Figure 4). Some examples of possible candidates identified according to these criteria would include *DHAR2*, *GSTU8*, *GSTU24*, and *GSTU25*. Interestingly, the proteins encoded by these genes are cytosolic (Figure 2), even though the initial location of oxidant generation in several of the studies shown in Figure 4 is expected to be mainly peroxisomal (*cat2*) or chloroplastic (paraquat, *flu*). The potential importance of cytosolic pathways in determining glutathione status in *cat2* has been previously noted (Mhamdi et al., 2010b).

### Genetic studies

While the data discussed above provide useful information on biochemical properties and expression patterns, establishing the physiological importance of specific components will require other approaches such as reverse genetics. Gene-specific loss-of-function studies will be required to establish the contributions of particular enzymes to GSSG production. As yet, there is relatively little information on the response of glutathione pools in plants specifically deficient in the genes discussed above. A few studies in Arabidopsis have generated potentially relevant data. A preliminary analysis of a *dhar2* mutant reported that although DHA was somewhat increased compared to Col-0, GSSG levels were similar in the two lines (Yoshida et al., 2006). Loss of *PRXIIF* function had slight effects on root glutathione pools in control conditions, but little difference from wild-type was observed during stresses involving exposure to cadmium or to an inhibitor of the mitochondrial alternative oxidase (Finkemeier et al., 2005). Knockout Arabidopsis *gstu17* mutants that showed altered stress responses were reported to have increased root and shoot glutathione contents in unstressed conditions, although no data for glutathione redox state or glutathione contents during stress were presented (Chen et al., 2012).

While loss-of-function studies are a more incisive approach to establishing the roles of specific genes, overexpression studies can also provide useful indications of the potential importance of a given enzyme. Several studies have overexpressed genes encoding GSTs, GPX, or DHAR, often with the objective of improving plant stress tolerance. A comprehensive discussion of these studies is beyond the scope of the present review. Hence, we limit ourselves here to reports that have included effects on glutathione status in the overexpressing lines.

Tobacco knockdown and overexpressor DHAR lines showed a substantial decrease and increase in extractable foliar activity, respectively (Chen and Gallie, 2005). While the knockdown lines had decreased ascorbate and increased DHA, the opposite effect was observed in the overexpressors (Chen and Gallie, 2005). The GSH:GSSG ratio was lower in the knockdowns and higher in the overexpressors, with overexpression increasing GSH:GSSG from about 4 in the control plants to almost 20, an effect largely due to increased GSH rather than decreased GSSG (Chen and Gallie, 2005). Another study in tobacco also reported that overexpression of Arabidopsis *DHAR2* increased the reduction state of the ascorbate pool, an effect that was observed both in control conditions and in response to aluminum stress (Yin et al., 2010). However, no difference in glutathione redox state between the control and overexpressors was observed in either condition. The authors concluded that GSH was not limiting for DHAR activity (Yin et al., 2010). Arabidopsis lines homologously overexpressing DHAR have also been described (Wang et al., 2010). Alongside improved stress tolerance, these plants had increased total pools of ascorbate and glutathione, both in control and stress conditions (high temperature, paraquat; Wang et al., 2010).

The picture that emerges from these studies is complex. If the reaction is considered in isolation, a negative relationship between the GSH:GSSG ratio and DHAR activity would be predicted, because the enzyme consumes GSH and produces GSSG. So far, there is little evidence that this relationship is observed in plants with genetically altered DHAR capacity. The reported increases in total glutathione and GSH:GSSG in tobacco and Arabidopsis overexpressor lines could be partly related to the need for enhanced GSH to support increased DHAR activity (Chen and Gallie, 2005; Wang et al., 2010). Thus, the effects of genetic manipulation of DHAR may not be limited to direct effects on GSH:GSSG ratios, e.g., because the plant may also respond by increasing glutathione synthesis. Another complication could be that altering the capacity of a single antioxidative enzyme may produce indirect effects on ROS availability that then alter the flux through other pathways that oxidize GSH, thus masking more direct effects.

Glutathione status was also assessed in tobacco lines overexpressing a GST with GSH peroxidase activity (Roxas et al., 2000). These lines showed enhanced tolerance to salt. Both in control and salt-stressed conditions, GST overexpression caused a more than three-fold increase in GSSG relative to control plants. This study therefore revealed that enhanced GST expression, which is a feature of oxidative stress responses (Figure 4), is able to decrease the reduction state of glutathione *in vivo*. It also reported the operation of secondary effects within the antioxidant system. Increased GSSG was associated with enhanced activities of ascorbate-glutathione pathway enzymes such as APX and MDHAR (Roxas et al., 2000).

## Modifications of glutathione status associated with GSSG accumulation

Why does GSSG accumulate during oxidative stress? Given the existence of opposing GSH oxidation and GSSG reduction activities, such accumulation is unlikely to be a simple result of conversion of GSH to GSSG. Rather, it is probably more accurately viewed as the net outcome of oxidation outpacing reduction, even if only slightly. As a simple hypothetical example: if enhanced H_2_O_2_ drives oxidation of 2 GSH to GSSG at 20 nmol.g^−1^FW min^−1^ (which even if one considers only DHAR as a source of GSSG is no more than 1% of typical capacities measured *in vitro*) but the *in vivo* GR activity is 1% slower (19.8 nmol.g^−1^FW min^−1^; about 2% of typical *in vitro* capacities), the two rates would entail a net accumulation of about 0.3 μmol.GSSG g^−1^ FW in 24 h. GSSG accumulation of this magnitude can be observed in catalase-deficient plants following transfer to oxidative stress conditions. The above calculation is obviously simplistic as the rates of the two reactions will vary as a function of changes in substrate concentrations following the onset of stress, and kinetic modeling would be required to examine the question more closely. It is intended merely to illustrate that slightly lower activity of GSSG reduction compared to GSH oxidation may be one factor driving a drop in the GSH: GSSG ratio *in vivo*.

In many cases, stress-induced GSSG accumulation in plants does not occur at the expense of decreased GSH. Rather, the GSH pool remains rather constant while even marked increases in total glutathione are almost entirely due to the accumulation of GSSG (Smith et al., 1985; Willekens et al., 1997; Mhamdi et al., 2010a). Available data suggest that this involves at least two processes additional to redox cycling between GSH and GSSG. The first is increased GSH neosynthesis as a result of processes that probably involve activation of cysteine and glutathione production at transcriptional and post-translational levels (Bick et al., 2001; Hicks et al., 2007; Gromes et al., 2008; Queval et al., 2009). The second is a marked change in glutathione compartmentation, with GSSG accumulation occurring particularly in the vacuole (Queval et al., 2011). This second process means that the GSH:GSSG ratio measured in tissue or whole cell extracts does not report on the actual glutathione status in specific compartments but is rather a composite value of GSH:GSSG ratios, which may differ widely between different subcellular locations (Noctor et al., 2013). For example, the cytosolic glutathione pool may well be less oxidized than that measured in extracts because a substantial amount of the GSSG generated by oxidative stress is shipped to the vacuole, possibly by ABCC transporters (Martinoia et al., 1993; Lu et al., 1998). This does not necessarily invalidate the GSSG:GSH ratio as a useful oxidative stress marker, because accumulation at sites such as the vacuole is predicted to be dependent on GSSG accumulation in other compartments caused by the oxidant-driven imbalance discussed above.

**Figure 5:**
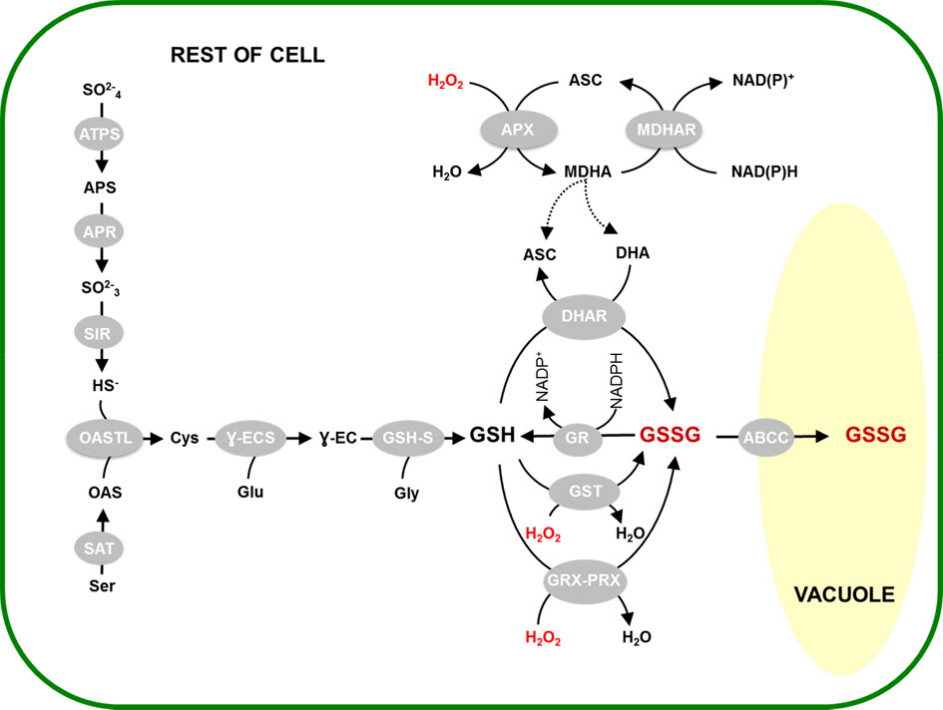
**Activation of glutathione synthesis and vacuolar sequestration linked to GSSG accumulation**. H_2_O_2_-triggered oxidation of GSH is considered to occur through the activities of DHAR (top), GST (center) or GRX-PRX (bottom). This is accompanied by activation of glutathione synthesis and GSSG sequestration in the vacuole, through the reactions shown on the left and right, respectively. Abbreviations as in Figure 1 legend, or as follows: ATPS, ATP sulphurylase; APS, adenosine phosphosulfate; APR, adenosine phosphosulphate reductase; SIR, sulphite reductase; OAS, *O*-acetylserine; OASTL, O-acetylserine(thiol)lyase; SAT, serine acetyltransferase; Ɣ-ECS, Ɣ-glutamylcysteine synthetase; GSH-S, glutathione synthetase; ABCC, sub-class C of the ATP-binding cassette transporters.

Figure 5 presents an overview that attempts to integrate some of the different processes that are likely to be involved in oxidative stress-driven changes in glutathione status. As well as GR activity, oxidation by the three main pathways we have discussed in this review is situated within the context of glutathione synthesis and the vacuolar sequestration of a substantial part of the GSSG that is generated. It is noteworthy that the vacuole is not the only compartment in which GSSG is highly enriched during oxidative stress. Marked accumulation of GSSG also seems to occur in the chloroplast. This has been reported in two independent studies of catalase-deficient plants using different techniques, despite the fact that the initial increase in H_2_O_2_ availability in these systems is expected to be extra-chloroplastic (Smith et al., 1985; Queval et al., 2011). While GSSG import from the cytosol cannot be completely discounted, the most influential process may be oxidation of GSH within the chloroplast (Noctor et al., 2013). Whatever the mechanisms, oxidation of GSH and/or accumulation of GSSG in the chloroplast may have consequences for thiol-dependent reactions in this compartment. These include regulation of chloroplast proteins by *S*-glutathionylation reactions or the TRX system (Dixon et al., 2005; Michelet et al., 2005; Zaffagnini et al., 2012) as well as activation of synthesis pathways that contribute to the accumulation of total glutathione in these conditions (Figure 5).

## Conclusions and perspectives

The post-genomics era has witnessed a dramatic increase in our understanding of plant antioxidative systems. Work over the last decade has also underscored their complexity. The present discussion has attempted to emphasize that a multiplicity of reactions may contribute to GSH oxidation during oxidative stress, leading to modifications in the status of this potentially important cellular redox signal. Whatever the reactions involved, the resulting changes in GSH:GSSG triggered by oxidants such as H_2_O_2_ may be signaled to sensitive proteins by catalysts such as certain GRX. Alternatively, the oxidation of certain thiol-dependent peroxidases, including some we have mentioned here, may itself act as a signal or signal relay independent of “bulk-phase” changes in GSH:GSSG.

While DHAR function in the ascorbate-glutathione pathway remains an outstanding candidate as a GSSG-generating enzyme, data from biochemical, transcriptomic and reverse genetics studies all suggest that other enzymes may contribute. The potential for redundancy between different enzyme classes is evident. It is also possible that considerable genetic redundancy exists within enzyme classes, both in peroxide removal and in GSH oxidation. This is most obviously apparent for the large GST family. Moreover, identification of the enzymes specifically involved in GSH oxidation using targeted loss-of-function studies may be complicated by the existence of different pathways that are able to replace or compensate for one another.

Despite these complexities, establishing the importance of the pathways able to generate GSSG from GSH *in planta* should be favored by the wide range of gene-specific mutants now available in Arabidopsis and other species. Kinetic modeling could also be useful to evaluate interactions between different reactions, and to define the limits of a system in which numerous components may act in parallel. A key issue could be (sub)cellular compartmentation, both of the reactions that cause GSH oxidation and of the GSSG that accumulates as a result. GSH-oxidizing processes may be condition-specific, with some reactions being more important in certain stresses than in others. Other issues that will need to be taken into account are the stage of plant development as well as modulating environmental conditions such as photoperiod, factors that may impose different patterns of gene expression in response to oxidative stress.

### Conflict of interest statement

The authors declare that the research was conducted in the absence of any commercial or financial relationships that could be construed as a potential conflict of interest.

## Abbreviations

a: GSH and GSSG are used here to refer specifically to the thiol and disulphide forms of glutathione. Where both forms may be referred to, “glutathione” is used.
b: GPX is used to refer to a specific family of genes encoding enzymes that may catalyse only low rates of this activity *in vivo*. To avoid confusion, the biochemical activity of glutathione peroxidation (e.g., catalysed by some glutathione *S*-transferases) is denoted by the abbreviation “GSH peroxidase.”
