# Supplementary material for: Missing links in understanding redox signaling via thiol/disulfide modulation: how is glutathione oxidized in plants?
Source: Front Plant Sci. 2013 Nov 25;4:477. doi: 10.3389/fpls.2013.00477 (PMC3838956; doi:10.3389/fpls.2013.00477)
Supplement: Supplementary file 1 [file DataSheet1.PDF]

Supplemental Table 1. List of Arabidopsis genes encoding candidates for oxidizing glutathione. DHA, dehydroascorbate. GPX, glutathione peroxidase. GSNO(R), *S*-nitrosoglutathione (reductase). GST, glutathione *S*-transferase. ROOH, peroxide. Chp, chloroplast. Cyt, cytosol. CW, cell wall. Mit, mitochondrion. Nuc, nucleus. Per, peroxisome. PM, plasmamembrane. Vac, vacuole.

Enzyme	AGI code	Oxidant	Subcellular localization
Dehydroascorbate reductase		DHA	
DHAR1	At1g19570		Per[14]
DHAR2	At1g75270		Cyt[6]
DHAR3	At5g16710		Chp/Mit[6,7]
Glutaredoxin-dependent peroxiredoxin		H_2O_2, ROOH	
GRX-PRXIIA	At1G65990		Not expressed[15]
GRX-PRXIIB	At1G65980		Cyt[17]
GRX-PRXIIC	At1G65970		Cyt[17]
GRX-PRXIID	At1G60740		Cyt[17]
GRX-PRXIIE	At3g52960		Chp[17]
GRX-PRXIIF	At3g06050		Mit[17]
Glutathione S-transferase		ROOH	
GSTF2	At4g02520		PM[16] /Cyt[7]/Chp[19]/Vac[4]
GSTF3	At2g02930		Cyt[*,18] / PM[2]
GSTF4	At1g02950		Cyt[*,18]
GSTF5	At1g02940		Cyt[*,18] /Mit[8]
GSTF6	At1g02930		Cyt[7]/ Mit[8]/Vac[4]
GSTF7	At1g02920		Cyt[11]/ Nuc[11]/Vac[4]
GSTF8	At2g47730		Cyt[7]/Chp[19]
GSTF9	At2g30860		Cyt[7]/Chp[19]/Vac[4]
GSTF10	At2g30870		Cyt[9]/Chp[19]/Vac[4]/CW[1]
GSTF11	At3g03190		Cyt[*,18]
GSTF12	At5g17220		Cyt[7]
GSTF13	At3g62760		Cyt[*,18]
GSTF14	At1g49860		Cyt[*,18]
GSTU1	At2g29490		Cyt[*,18]
GSTU2	At2g29480		Cyt[7]
GSTU3	At2g29470		Cyt[*,18]
GSTU4	At2g29460		Cyt[*,18]
GSTU5	At2g29450		Cyt[9] /PM[12]
GSTU6	At2g29440		Cyt[9]
GSTU7	At2g29420		Cyt[7]
GSTU8	At3g09270		Cyt[*,18]
GSTU9	At5g62480		Cyt[7]
GSTU10	At1g74590		Cyt[*,18]
GSTU11	At1g69930		Cyt[7]
GSTU12	At1g69920		Nuc[7]
GSTU13	At1g27130		Cyt[18]
GSTU14	At1g27140		Cyt[*,18]

GSTU15	At1g59670		Cyt[*,18]
GSTU16	At1g59700		Cyt[*,18]
GSTU17	At1g10370		Cyt[*,18] /Chp[19]
GSTU18	At1g10360		Cyt[*,18]
GSTU19	At1g78380		Cyt[7] /Chp[13,19]/ PM[12]/Vac[10]
GSTU20	At1g78370		Cyt[*,5,18]/Chp[19]/Nuc[5]/apoplast[3]
GSTU21	At1g78360		Cyt[18]
GSTU22	At1g78340		Cyt[18]/Nuc[*]
GSTU23	At1g78320		Cyt[18]
GSTU24	At1g17170		Cyt[18]
GSTU25	At1g17180		Cyt[18]
GSTU26	At1g17190		Cyt[18]
GSTU27	At3g43800		Cyt[18]
GSTU28	At1g53680		Cyt[7]
GSTL1	At5g02780		Cyt[7]
GSTL2	At3g55040		Chp[6]/Cyt[7]/Per[7,19]
GSTL3	At5g02790		Cyt[7]
GSTT1	At5g41210		Per[7]
GSTT2	At5g41240		Per[7]
GSTT3	At5g41220		Per[7]
GSTT3L[(with Myb-like extension)]	At5g41220		Nuc[7]
GSNO reductase		GSNO	
GSNOR	At5g43940		Cyt[9]
Glutathione peroxidase[15]		ROOH, H_2O_2	
GPX1	At2g25080		Chp[**]
GPX2	At2g31570		Cyt[**]
GPX3	At2g43350		Mit[**]
GPX4	At2g48150		Cyt[**]
GPX5	At3g63080		ER, Cyt[**]
GPX6	At4g11600		Chp[**]
GPX7	At4g31870		Chp, Per[**]
GPX8	At1g63460		Cyt[**]

The table summarizes subcellular localization. Data was compiled on the basis of available literature, referenced below.

1. Bayer, E.M., Bottrill, A.R., Walshaw, J., Vigouroux, M., Naldrrett, M.J., Thomas, C.L., and Maule, A.J. (2005). Arabidopsis cell wall proteome defined using multidimensional protein identification technology. *Proteomics*, 6, 301-311.

2. Benschop, J.J., Mohammed, S., O'Flaherty, M., Heck, A.J., Slijper, M., Menke, F.L. (2007) . Quantitative phosphoproteomics of early elicitor signaling in Arabidopsis. *Mol Cell Proteomics.* 6,1198-1214.

3. Bindschedler, L.V., Palmblad, M., Cramer, R. (2008) Hydroponic isotope labelling of entire plants (HILEP) for quantitative plant proteomics; an oxidative stress case study. *Phytochemistry.* 69,1962-1972.

4. Carter, C., Pan, S., Zouhar, J., Avila, E.L., Girke, T., and Raikhel, N.V. (2004). The vegetative vacuole proteome of *Arabidopsis thaliana* reveals predicted and unexpected proteins. *Plant Cell* 16: 3285-3303.

5. Chen, I.C., Huang, I.C., Liu, M.J., Wang, Z.G., Chung, S.S., Hsieh, H.L. (2007) .Glutathione S-transferase interacting with far-red insensitive 219 is involved in phytochrome A-mediated signaling in Arabidopsis. *Plant Physiol.* 143:1189-1202

[6.]Dixon, D.P., Davis, B.G., Edwards, R. (2002). Functional divergence in the glutathione transferase superfamily in plants: identification of two classes with putative functions in redox homeostasis in *Arabidopsis thaliana*. *J. Biol. Chem*. 277, 30859–30869.

[7.]Dixon, D.P., Hawkins, T., Hussey, P.J., and Edwards, R. (2009). Enzyme activities and subcellular localization of members of the Arabidopsis glutathione transferase superfamily. *J. Exp. Bot.* 60, 1207–1218.

[8.]Heazlewood, J.L., Tonti-Filippini, J.S., Gout, A.M., Day, D.A., Whelan, J., Millar, A.H. (2004). Experimental analysis of the arabidopsis mitochondrial proteome highlights signaling and regulatory components, provides assessment of targeting prediction programs, and indicates plant-specific mitochondrial proteins. *Plant Cell* 16, 241–256.

[9.]Ito, J., Batth, T.S., Petzold, C.J., Redding-Johanson, A.M., Mukhopadhyay, A., Verboom, R., Meyer, E.H., Millar, A.H., Heazlewood, J.L. (2011). Analysis of the Arabidopsis cytosolic proteome,highlights subcellular partitioning of central plant metabolism. *J Proteome Res.* 10, 1571-1582.

[10.]Jaquinod, M., Villiers, F., Kieffer-Jaquinod, S., Hugouvieux, V., Bruley, C., Garin, J., Bourguignon, J. (2007) A proteomics dissection of *Arabidopsis thaliana* vacuoles isolated from cell culture. *Mol Cell Proteomics.* 6, 394-412.

[11.]Koroleva, O. A., Tomlinson, M. L., Leader, D., Shaw, P., and Doonan, J. H. (2005). High-throughput protein localization in Arabidopsis using Agrobacterium-mediated transient expression of GFP-ORF fusions. *Plant J.* 41, 162–174.

[12.]Marmagne, A., Ferro, M., Meinnel, T., Bruley, C., Kuhn, L., Garin, J., Barbier-Brygoo, H. and Ephritikhine, G. (2007) A high content in lipid- modified peripheral proteins and integral receptor kinases features in the arabidopsis plasma membrane proteome. *Mol. Cell Proteomics* , 6 , 1980– 1996.

[13.]Peltier, J.B., Cai, Y., Sun, Q., Zabrouskov, V., Giacomelli, L., Rudella, A., Ytterberg, A.J., Rutschow, H., and van Wijk, K.J. (2006). The oligomeric stromal proteome of Arabidopsisthaliana chloroplasts. *Mol. Cell Proteomics*. 5, 114–133.

[14.]Reumann, S., Quan, S., Aung, K., Yang, P., Manandhar-Shrestha, K., Holbrook, D., Linka, N., Switzenberg, R., Wilkerson, C.G., Weber, A.P., Olsen, L.J., Hu, J. (2009). In- depth proteome analysis of Arabidopsis leaf peroxisomes combined with in vivo subcellular targeting verification indicates novel metabolic and regulatory functions of peroxisomes. *Plant Physiol.* 150, 125–143.

[15.]Rouhier, N., Jacquot, J.P. (2005). The plant multigenic family of thiol peroxidases. *Free Radic. Biol. Med.* 38, 1413-1421.

[16.]Smith, A.P., Nourizadeh, S.D., Peer, W.A., Xu, J., Bandyopadhyay, A., Murphy, A.S., and Goldsbrough, P.B. (2003). Arabidopsis AtGSTF2 is regulated by ethylene and auxin, and encodes a glutathione S-transfer- ase that interacts with fl avonoids. *Plant J.* 36, 433-442.

[17.]Tripathi, B.N, Bhatt, I., and Dietz, K.J. (2009). Peroxiredoxins: a less studied component of hydrogen peroxide detoxification in photosynthetic organisms. *Protoplasma* 235, 3–15.

[18.]Wagner, U., Edwards, R., Dixon, D.P., Mauch, F. (2002) Probing the diversity of the Arabidopsis glutathione S-transferase gene family. *Plant Mol Biol*. 49, 515-532.

[19.]Zybailov, B., Rutschow, H., Friso, G., Rudella, A., Emanuelsson, O., Sun, Q., van Wijk, K.J. (2008). Sorting signals, N-terminal modifications and abundance of the chloroplast proteome. *PLoS ONE* 3, e1994 1–19.

[*]AtSubP analysis from TAIR (2012-08-23)

[**]Localizations of Gpxs are essentially based on prediction programs (Psort (http://www.psort.nibb.ac.jp/form.html), TargetP (http://www.cbs.dtu.dk/services/TargetP/)).

Supplemental Table 2. Summary details of studies from which the expression data shown in Figure 4 were obtained. All data were extracted from www.genevestigator.com (Hruz et al., 2008). Where data were deposited in relation to a specific original publication, these are indicated.

Reference	Treatment
Davletova et al., 2005	hydrogen peroxide: Col-0 seedlings (5-day-old) were treated with 20 mM H_2O_2 for 1 hour. The controls were not treated.
www.genevestigator.com	ozone: Col-0 seedlings (cotyledons fully opened) were treated with 500ppb ozone for 6 hours. The controls were air-treated
www.genevestigator.com	paraquat: Col-0 plants were grown for 2 weeks (16h light (120 µmol m−2 s−1) / 8h dark cycles, 23°C, 65–70% relative humidity) and then removed from soil and floated with adaxial surfaces facing the light source in 50µM MV, 0.01 % (v/v) Tween 20 solution for 2h at 120 µmol m^{-2} s^{-1} illumination, 23°C. After MV treatment aerial parts were separated from roots. The controls were treated with 0.01 % (v/v) Tween 20 solution for 2h.
Laloi et al., 2007	*flu* mutant: Col-0 and the *flu* mutant were grown in long days for 3 weeks and harvested after and re-illuminated for 120 min following the dark period.
Mhamdi et al., 2010	*gr1*, *cat2* and *cat2 gr1* mutants: plants were grown for 21 days at high CO2 (8h light /16h dark regime) and then transferred to air for 4 days.
Queval et al., 2012	*cat2* mutants: plants were grown for 5 weeks at high CO2 (8h light /16h dark regime) and then transferred to air for 2 or 4 days

Davletova, S., Schlauch, K., Coutu, J., and Mittler, R. (2005). The zinc-finger protein Zat12 plays a central role in reactive oxygen and abiotic stress signaling in Arabidopsis. *Plant Physiol.* 139, 847-856.

Hruz, T., Laule, O., Szabo, G., Wessendorp, F., Bleuler, S., Oertle, L., Widmayer, P., Gruissem, W., and Zimmermann, P. (2008). Genevestigator V3: a reference expression database for the meta-analysis of transcriptomes. Advances in Bioinformatics 420747

Laloi, C., Stachowiak, M., Pers-Kamczyc, E., Warzych, E., Murgia, I., and Apel, K. (2007). Cross-talk between singlet oxygen- and hydrogen peroxide-dependent signaling of stress responses in Arabidopsis thaliana. *Proc. Natl. Acad. Sci.* USA 104: 672-677.

Mhamdi, A., Hager, J., Chaouch, S., Queval, G., Han, Y., Taconnat, Y., Saindrenan, P., Issakidis-Bourguet, E., Gouia, H., Renou, J.P., and Noctor, G. (2010). *Arabidopsis* GLUTATHIONE REDUCTASE 1 is essential for the metabolism of intracellular H_2O_2 and to enable appropriate gene expression through both salicylic acid and jasmonic acid signaling pathways. *Plant Physiol.* 153,1144-1160

Queval, G., Neukermans, J., Vanderauwera, S., Van Breusegem, F., and Noctor, G. (2012). Day length is a key regulator of transcriptomic responses to both CO_2 and H_2O_2 in Arabidopsis. *Plant Cell Environ.* 35, 374-387.
